# Intersecting Structural Barriers and Hypertension Management Among Middle East and North Africa Refugees Resettled in the United States: A Qualitative Study

**DOI:** 10.1177/10901981251358716

**Published:** 2025-10-07

**Authors:** Dania Abu Baker, Job Godino, Behnan Albahsahli, Becky Marquez, Tala Al-Rousan

**Affiliations:** 1 University of California, San Diego, La Jolla, CA, USA; 2 San Diego State University, San Diego, CA, USA; 3 Family Health Centers of San Diego, San Diego, CA, USA

**Keywords:** social determinants of health, health equity, health disparities, hypertension, refugee

## Abstract

Hypertension remains one of the leading causes of morbidity and mortality worldwide, affecting more than 1.28 billion people. Refugees have almost twice the risk of cardiovascular disease compared to native populations and other immigrants due to uncontrolled hypertension. Evidence on aspects of structural oppression that impact hypertension management and the experiences of minority populations in managing their hypertension post-resettlement remains lacking especially in the case of refugees whose number is increasing dramatically due to mass displacement. We explored the intersecting structural oppression impacting hypertension management among refugees using in-depth interviews with 54 Syrian and Iraqi refugees who were resettled in California. Structural oppression risk factors that were identified to impact hypertension management were (a) structural barriers (language barriers, lack of health insurance in transit countries), (b) social stigmatization (social isolation, family separation, discrimination), (c) economic inequities (living in poverty, low-pay and high-risk jobs), and (d) discriminatory policies (refugee ban). Our findings highlight that structural oppression affects hypertension management among refugees post-resettlement, highlighting the need for tailored interventions and policy changes that take these factors into account to promote hypertension management in this population to prevent health disparities.

Hypertension (HTN) affects 1.28 billion people worldwide, with nearly half of the adults in the United States having uncontrolled HTN according to the World Health Organization. Uncontrolled HTN is a risk factor for congestive heart failure, stroke, coronary artery disease, and intracerebral hemorrhage. This is a major public health burden as uncontrolled HTN accounts for 10.4 million deaths per year (Unger et al., 2020). Uncontrolled HTN is more prevalent in low and middle-income countries where most refugees in the United States come from (Okati-Aliabad et al., 2022). The number of individuals with HTN in low and middle-income countries is 1.04 billion compared to 349 million in high-income countries. The Middle East is a region in which HTN is increasing at an alarming rate, with an estimated prevalence of 24.4% (Okati-Aliabad et al., 2022).

The Middle East and North African region (MENA) has experienced political unrest for the past 70 years and became the main producer of refugees globally; over 25% of total global refugee population comes from Syria according to the United Nations Higher Commissioner for Refugees (UNHCR, 2023a, 2023b). The mass displacement from Arab countries like Syria has been referred to as the worst man-made disaster since World War II by the United Nations (UNHCR, 2023a). A refugee is someone who has been forced to flee their country to escape armed conflict, persecution, or generalized violence. There are around 9.2 million Iraqis internally displaced or refugees abroad, while 6.8 million Syrians are refugees abroad and are internally displaced (UNHCR, 2023b). Refugees are commonly exposed to structural oppression. In the context of public health, structural oppression refers to the societal factors and institutional mechanisms that create and perpetuate health disparities and inequities among different population groups based on social determinants such as race, ethnicity, socioeconomic status, gender, and other intersecting identities. Refugees experience structural oppression such as interrupted health care access and social stigmatization associated with chronic stress and poor mental health outcomes which may all impact chronic disease management (Bautista et al., 2019). For example, those with high hair cortisol, a biological marker of chronic psychosocial stress, were twice more likely to be hypertensive than those with low hair cortisol (Bautista et al., 2019). However, the ways by which structural oppression contributes to the clustering of psychosocial stressors with HTN among MENA refugees are still undocumented in research.

The clustering of two health conditions, such as HTN and psychosocial stress, can be understood through a syndemics framework. Syndemics theory argues that different risk factors interact to yield adverse health outcomes, and that the joint interaction of these factors yields a larger disease burden than the sum of risk factors in isolation (Singer & Clair, 2003). These factors are intertwined and affect each other, thus examining them individually as distinct factors will not produce an accurate understanding of the health problem at hand. In the case of MENA refugees resettled in the United States, these risk factors are forms of structural oppression. This model examining different forms of structural oppression as risk factors provides a holistic approach to improve the care and treatment for those with chronic illness, such as HTN. HTN is the most prevalent noncommunicable disease among Syrian and Iraqi refugees (Al-Rousan et al., 2022). One study found that HTN prevalence is higher among Syrian refugees in Turkey than among individuals living in pre-war Syria (Denli Yalvac, 2020), an observation that was also seen among Iraqi refugees resettled in the United States (Kumar et al., 2021). Refugees are also at higher risk of HTN than the general population where they resettled (Zibara et al., 2021). This raises questions about certain structural oppression after refugees are resettled and on the epidemiology of HTN in displaced populations.

To date, there is scarce literature evaluating the role of different forms of structural oppression on HTN management profiles (medical adherence such as taking medications, attending medical appointments, and self-monitoring of blood pressure levels) of MENA refugees in the United States. Particularly, these refugees top the list of the fastest-growing foreign-born population in San Diego, California (Wank & Sanchez, 2020). There is evidence of increased HTN prevalence among refugees (Al-Rousan et al., 2022) and deteriorating health among this population post resettlement (CDC, 2023). However, they remain one of the most understudied populations suffering health disparities that linger over generations of Americans (Al-Rousan et al., 2022). The aim of this study is to examine the syndemic risk factors that impact HTN/stress syndemic in MENA refugees in the United States.

## Methods

### Community-Based Participatory Research Study Design

A Community-Based Participatory Research (CBPR) partnership was formed in July 2019 between the Displacement and Health Research Center at UCSD and Majdal Community Center. The research team included UCSD graduate students who were refugees or second-generation immigrants as well as members from the Majdal Community Center. We formed a community advisory board that included Arab refugee patients diagnosed with HTN who received care at the Family Health Center, a federally qualified health clinic, and community leaders from Majdal Community Center. The research team and community advisory board jointly designed this research study, including an interview guide, and discussed results collaboratively to ensure accurate representation and reflection of the community’s narrative.

### Recruitment and Data Collection

The institutional review board (IRB) at the University of California San Diego approved this research (#200063). All participants provided written informed consent. In this qualitative study, we used semi-structured interviews with Syrian and Iraqi refugees in San Diego to explore the syndemic interactions of structural oppression impacting HTN management. Participants included 54 MENA refugees (23 Syrians and 31 Iraqis) in San Diego. The inclusion criteria were (a) having been admitted in the United States on a refugee status from Iraq or Syria, (b) being 18 years old or older with a confirmed clinical diagnosis of HTN, and (c) being able to give informed consent. Recruitment of our convenient sample was facilitated through collaboration with Majdal Community Center and Family Health Center. Participants were contacted via phone through a patient/beneficiaries’ list provided by our collaborators and were provided detailed information on the research aims. A certified translator from the community translated the research material. Interviews were conducted by bilingual (English and Arabic) interviewers from the study research team who were trained to conduct semi-structured interviews. Each interview lasted for around 45–60 minutes and was audio-recorded following the participant’s permission. Interviews were transcribed and translated by a certified bilingual translator.

We collected demographic characteristics at the start of the interviews. These included age, gender, country of origin, marital status, educational achievement, employment status, annual household income, and English proficiency. The interview guide was developed after having conducted a scoping review of the literature on HTN management in low and middle-income countries, more specifically among MENA refugees and incorporating advice from the community advisory board. The interview guide was modified iteratively based on feedback from participants. Questions focused on exploring practices and barriers related to HTN management, impact of refugee journey and structural oppression on HTN management, and impact of policies on health care.

### Qualitative Analysis

We utilized inductive thematic analysis to analyze the data. Thematic analysis followed Braun and Clarke’s six-phase framework (Braun & Clarke, 2006). The qualitative analysis program Nvivo 12.0 was used to manage the codes (Nvivo, 2002). Three members of our team collaboratively coded five transcripts and created a codebook. All coders used the codebook as a reference for final codes. All coders then met with refugee community advisory board members to discuss their codes and categorized similar codes into themes. Any discrepancies in codes were resolved based on consensus during research team meetings. Thematic saturation was reached when no new codes emerged, yet all transcripts were coded. Codes were grouped together, according to their similarities, to form an overarching theme. The essence of each theme was identified by coders and used to provide names to each theme. The coders collaboratively selected several quotes that they agreed captured the main points of each theme. These quotes were later used when reporting the results of the research.

## Results

**Figure 1.**
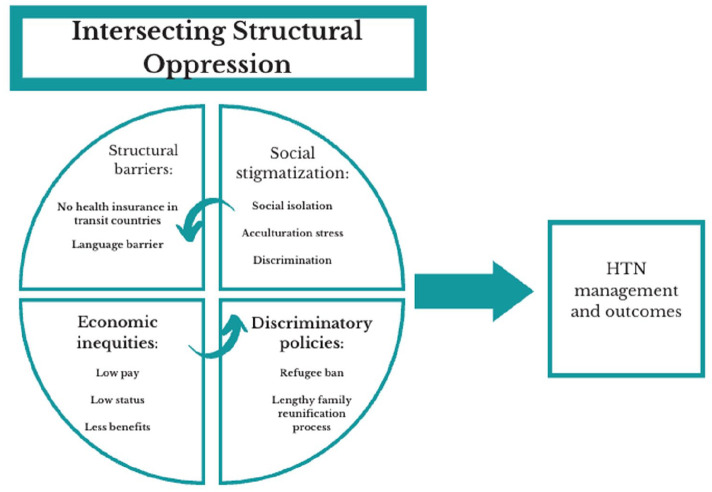
Syndemic Interactions of Structural Oppression Impacting HTN in MENA Refugees. *Note*. HTN = hypertension; MENA = Middle East and North African region.

Demographic characteristics are presented in Table 1. The majority of the sample were males (57.4%), married (87.0%), with a highest educational level below a high school degree (38.9%), unemployed (87.0%), and not proficient in English (68.5%). The median age of the sample was 59.0 ± 10.3. Four themes emerged as factors impacting HTN management. Our team identified themes and subthemes relevant to our research goals and organized them based on the syndemics theory (Figure 1).

**Table 1.** Demographics Characteristics of Refugee Sample (n = 54).

Demographic	Iraqi (*n* = 31)	Syria (*n* = 23)	All (*n* = 54)
Age, (median, *SD*) in years	64.0 (10.9)	54.4 (7.8)	59.0 (10.3)
Age, range in years	38–76	38–69	38–76
Years in the US (*Mdn*, *SD*)	8.0 (6.2)	6.0 (1.3)	6 (5.1)
1–3	-	1 (4.3)	1 (1.9)
4–6	11 (35.5)	18 (78.2)	29 (53.7)
7>	20 (64.5)	4 (17.4)	24 (44.4)
Gender			
Women	15 (48.4)	8 (34.8)	23 (42.6)
Men	16 (51.6)	15 (65.2)	31 (57.4)
Marital status			
Married	26 (83.9)	21 (91.3)	47 (87.0)
Widowed	5 (16.1)	2 (8.7)	3 (13.0)
Highest level of education			
Less than high school	8 (25.8)	13 (56.5)	21 (38.9)
High school	5 (16.1)	8 (34.8)	13 (24.1)
Vocational certificate	5 (16.1)	1 (4.3)	6 (11.1)
Bachelor’s degree	11 (35.5)	1 (4.3)	12 (22.2)
Employed			
Yes	5 (4.4)	2 (4.4)	7 (13.0)
No	26 (35.6)	22 (35.6)	47 (87.0)
Annual income			
Less than $15,000	13 (41.9)	17 (73.9)	30 (55.6)
$15,001–$25,000	11 (35.5)	5 (21.7)	16 (29.6)
$25,001–$35,000	5 (16.1)	-	5 (9.3)
$35,001–$50,000	2 (6.5)	-	2 (3.7)
$50,000 and above	-	1 (4.3)	1 (1.9)
Proficient in English			
Yes	13 (41.9)	4 (17.4)	17 (31.5)
No	18 (58.1)	19 (82.6)	37 (68.5)

*Note*. Values represent numbers and percentages unless indicated otherwise.

### Theme 1 From Interviews: Structural Barriers Impacting Hypertension Management

Refugees in this sample were exposed to various traumatic situations in their home countries. These include witnessing the kidnapping or killing of others, as well as witnessing airstrikes and bombings. This resulted in the loss of one’s home, belongings, and money. Exposure to such horrors was associated with worse mental health including fear, flashbacks, stress, and mourning. Others shared receiving a clinical diagnosis of depression and taking psychotropic medications.

It [the war] affected us a lot, we carry many memories and trauma with us, sadness is inevitable, we mourn our beloved ones and destroy our country. (Iraqi, male)

Refugees in this sample linked their HTN diagnosis and suboptimal disease management to the stress of war and displacement. In such traumatizing situations, it was difficult for those refugees to maintain their wellbeing and manage their HTN as they experienced chronic stress. The priority was to survive and ensure the safety of their loved ones. Refugees in our sample were unable to access health clinics and medication availability was limited, hence negatively impacting their HTN management. This structural oppression in the form of suboptimal quality and availability of care also left many refugees unaware of their HTN diagnosis until they arrived in host countries.

It [the war] exacerbated my HTN. Rockets were raining down on us and we were seeing our beloved ones dying in Aleppo. Above all that, I didn’t take any HTN medications during that time. (Syrian, male)

In terms of health care services in the United States, refugees in this sample described how the availability of interpreters facilitated communication with health care providers. However, participants conveyed a sense that despite the translators’ presence, their messages were not always fully delivered. There was a perception that certain subtleties and nuances were lost in translation, leading to a potential gap in conveying the depth and intricacies of their experiences. “The translator does not always deliver the right idea and information to me or what I am trying to tell my non-Arabic speaking doctors.” (Iraqi, female)

### Theme 2 From Interviews: Economic Inequities Exacerbating HTN

The traumatic situations that refugees in this sample experienced were not limited to those in their home countries. Even though they have arrived in safer war-free countries, they continue to face daily psychosocial stressors such as unemployment, financial difficulties, and discrimination. Refugees in this sample described how their status as asylum seekers in countries like Jordan and Turkey prevented them from obtaining jobs, which increased their stress levels. Furthermore, refugees were not eligible for health insurance, and they were unable to afford the expenses of private clinics and medications, which limited their access to health care services needed to manage their HTN.

To be honest, my health conditions deteriorated in Jordan. There was so much pollution that caused me asthma and mental stress from the low wages even though I worked so hard at the salon. I couldn’t afford medications over there I used to take my sister-in law’s Jordanian ID card to get the medications in Jordan, I would ride three buses to reach the place. (Syrian, female)

Refugees in this sample continued to experience these economic stressors even after arriving in the United States, thus exacerbating their mental health problems and HTN. Modest financial aid, as well as health insurance, was provided to newly arrived refugees for a limited amount of time. Afterwards they were left to navigate health and other systems on their own with limited English proficiency and resources to help them secure jobs, navigate the school systems for themselves and their children, and integrate into their new societies successfully. In addition, the language barrier prevented them from advancing in their careers and obtaining higher-paying jobs, thus making it difficult for them to afford the high living costs in the United States. These financial stressors contributed to worse mental health in refugees as they experienced chronic stress, and racism which in turn exacerbated their HTN outcomes.

When we came here [the U.S.] it was only for the first 1-3 months that we were taken care of and helped out. After that, we were all on our own and reality hit us hard. It is harder than we expected to live here and try to make it. (Iraqi, female)

### Theme 3 From Interviews: The Stress Resulting From Discriminatory Policies Exacerbating HTN Outcomes

The charged political climate including the U.S. Refugee Ban along with complicated immigration procedures prevented refugees from being reunited with their families.^20^ After completing necessary and long immigration paperwork, the refugee ban was implemented right when refugees were finally about to be reunited with family and prevented their reunification. Others described how this process involved years of waiting, only to be suddenly suspended. Refugees related the inability to be reunited with their loved ones with worse mental health. The uncertainty of when they will be reunited contributed to experiencing chronic stress, nightmares, not wanting to eat food or drink water for long periods which were related to deterioration in their overall health and increased hospital visits. This chronic stress also contributed to difficulties maintaining normal blood pressure levels.

It [the refugee ban] affected me so much, at that time my daughter was supposed to travel to the U.S and see us and this policy prevented her from visiting us. This drastically made me sicker and I started visiting the hospital more often. I was dizzy all the time and had nervous breakdowns.” (Syrian, female)

### Social Stigmatization Causing Chronic Stress

Refugees shared how their social life was negatively impacted after settling in the United States, impacting their social integration post resettlement. This was ascribed to the busy lifestyle in the United States where refugees spend most of their time at work to provide for their families. This social isolation was associated with symptoms of worse mental health, such as stress and loneliness. They lacked the social networks needed to communicate concerns and reduce stress. Social isolation also limited the amount of health information on resources available to refugees, such as the availability of Arabic-speaking doctors, thus impacting their ability to better manage their HTN.

There is a life in the Middle East and a social ambiance that de-stresses us and makes us happy . . . Life is more serious in America, you come back from work at 6 pm and then people are isolated and not socially active as in Iraq where you can find late night outings and fun. (Iraqi, male)

In addition to the social isolation that refugees in our sample experience in the United States, they also described how they constantly worry about family members abroad. These refugees were especially concerned about the family members they had to leave behind in a dangerous context and limited food security and livelihood in their home countries. This contributed to chronic stress and worse mental health outcomes among refugees in our sample, thus exacerbating their HTN outcomes.

Now, as you know, with all the losses we went through, both of my sons were wounded by the strikes, the demolition of my house. My children being away from me, I sit and eat and sleep while my children are suffering in Syria. My HTN was caused by all of this. I used to be a happy, positive person, but now I feel so negative. (Syrian, female)

My HTN started after I left Syria, I have lost a lot of loved ones, siblings, and my depression has caused my HTN. For example, I haven’t seen my siblings in almost 11 years. Whenever I get depressed and start crying my BP goes up. (Syrian, male)

## Discussion

In this study, we used a syndemics theory to explore structural oppression impacting HTN /stress syndemic in MENA refugees in the United States. We found that the traumatic experiences refugees have experienced contribute to chronic stress, which limited the ability to manage their HTN. Chronic stressors and economic inequities during their asylum-seeking journey contributed to further deterioration in mental health and HTN outcomes of MENA refugees. Structural challenges that refugees experience in the United States such as separation from family members due to discriminatory immigration policies contributed to increased psychological stress and, in turn, worse HTN management. Our study is the first to document intersecting structural oppression impacting HTN management among this vulnerable minority group.

Our findings highlight the negative impacts of psychological trauma on HTN management among MENA refugees. The types of traumatic situations that this population has endured are well documented in the literature. It is especially challenging to manage HTN in the context of humanitarian crises such as war (Zibara et al., 2021). Refugees in our sample described how they were unaware of their HTN diagnosis due to interrupted health care services in their home countries during conflict. Those who were aware of their diagnosis prioritized survival and providing basic needs for their family members over buying/taking their medications.

While fleeing the country of conflict provided safety for refugees, they continued to experience daily economic stressors. The lack of health insurance along with unaffordable private health care and medication expenses in transit countries such as Jordan all contributed to poor HTN management among refugees in our sample. For example, they were unable to afford health care services including medical checkups and medication. These challenges are consistent with the reported experiences of Liberian refugees in the United States (Ludwig & Reed, 2016). Such economic inequity is a result of employment discriminatory policies in transit countries, such as Jordan. Employment restrictions by the Jordanian government, such as requiring the purchase of work permits and limiting the sectors of work, led Syrian refugees to work informally. Informal work is often characterized by below minimum wage pay and harsh work conditions (Wells et al., 2016).

Refugees in this study continued to be exposed to economic stress even after settling in the United States. This is similar to previous accounts of African refugees resettled in the United States where refugees are restricted to pursuing low-payment jobs that do not require English proficiency (Kim et al., 2022). Unemployment, poverty, and limited integration in host communities are all considered chronic economic stressors which relate to poor HTN outcomes (Al-Rousan et al., 2022). Structural oppression in the form of economic inequities can be attributed to resettlement policies in the United States. During the initial 90-day period upon arrival, resettlement agencies collaborate with state and local governments as well as community organizations to assist resettled refugees in integrating into their communities. Despite the attainment of higher education degrees, refugees in the United States are often forced to take the first job opportunity they are offered to secure a source of income within the short 3-month arrival period. This leaves refugees with lower pay and benefit jobs compared to their occupation in their home country.

Social isolation, stigmatization, and difficulties integrating in the United States were also highlighted as structural oppression impacting HTN management among the interviewed refugees. They shared how most of their time was spent at work, leaving them with limited social lives. This experience of social disconnection was similarly shared by Cambodian refugees in previous research (Berthold et al., 2019). Social support is key to coping with adverse and stressful situations, and therefore, social isolation in the form of lack of emotional support and engagement in the community is highlighted as a risk factor for poor health and wellbeing outcomes. More specifically, research has revealed a significant association between social isolation and being diagnosed with cardiovascular disease within a 5-year follow-up period.

Refugees in our study also associated the exacerbation of their mental health problems and HTN to the discrimination they experience in the United States. Similar experiences were expressed previously by MENA refugee women who wore the hijab and therefore were visible targets for Islamophobic hate crimes (Murad & Versey, 2021). Discrimination is intertwined in the literature with acculturation stress in minority groups, which is a known risk factor for poor mental health and HTN through experiencing chronic stress (Tellechea & Pirola, 2017). Therefore, stricter policies are needed to protect refugees against discrimination, hate, and islamophobia.

The U.S. Refugee Ban, a policy that was passed by the Trump administration in December 2017, prevented many refugees from reuniting with their families (Pierce, 2017). This is consistent with the experiences of Afghan refugees in previous research (Mayo, 2018). The uncertainty of being reunited with family members contributed to chronic stress and in return worse HTN outcomes in refugees in our sample. They described how this stress translated into forgetting to take their medications, increased hospital visits, and severe distress in some cases. This was also evident in previous research on immigrants, in which family separation at the border was associated with poor mental health outcomes (Shadid & Sidhu, 2021). Furthermore, these discriminatory policies cultivated alienation and acculturation stress in MENA refugees (Murad & Versey, 2021). This is similar to findings of family separation policies at the border contributing to poor adherence to chronic disease management, reduced health-seeking behavior, and limited trust in the health care system (Callaghan et al., 2019). MENA refugees come from a collectivist culture in which social and economic resources were shared in the family unit; therefore, separation of family results in a disruption to this support network and in turn stress.

Our findings highlight the complexity of HTN/stress syndemic among MENA refugees resettled in the United States. Past psychological trauma and current structural oppression in the form of economic inequity, health care disparity, social stigmatization, and discriminatory policies contribute to poor HTN management and outcomes. Our findings inform the design of future multi-level health management services and interventions that address not only individual-level risk factors but also structural oppression. This will require collaboration between primary health care providers, mental health services, and social services. One example of such interventions is a piloted psychosocial intervention that examined common emotional experiences in refugees resettled in the United States and ways those refugees can use community resources to promote wellbeing. Results revealed significant reductions in emotional distress and an increase in self-efficacy. Current resettlement policies are brief and focus on rapid self-sufficiency. Public policy must promote economic and health equity among refugees, which will facilitate the integration of refugees in the United States. One way to achieve that is through livelihood training and English courses. The long-term implications of improving HTN management include better prognosis, reduced risk of heart and brain disease, and overall reduced medical costs. To reduce health disparity among refugee populations, researchers, health care providers, and policy makers must uproot intersecting structural oppression that drive inequities in health care access, economic opportunities, and social integration.

Although this study addresses a gap in the literature, it has some limitations. Participants were sampled from either a community-based organization or a health care center, which may limit generalizability to all refugees. However, both locations were selected because they are in the area where most MENA refugees reside and can represent both those who are connected to health care and those who are not. Researcher-induced bias is possible in inductive thematic analysis. However, the analysis of the themes by multiple members of the research team and regular meetings ensured that the themes were robust. Finally, our findings may not reflect the determinants of HTN management in refugee communities originating from other parts of the world since refugees may exhibit different HTN prevalence and management profiles. However, we believe that these findings may be applicable since most refugees face similar challenges along the migratory route and after resettling in the United States.
